# First Report on the Complete Genome Sequence of *Lactobacillus crustorum* MN047, a Potent Probiotic Strain Isolated from Koumiss in China

**DOI:** 10.1128/genomeA.00048-17

**Published:** 2017-03-23

**Authors:** Lanhua Yi, Xing Guo, Lian Liu, Chunge Shao, Xin Lü

**Affiliations:** College of Food Science and Engineering, Northwest A&F University, Yangling, Shaanxi Province, China

## Abstract

The complete genome sequence of* Lactobacillus crustorum* deciphered by PacBio RS II and Illumina HiSeq 4000 sequencing was first reported with one chromosome and two plasmids. Sequence analysis of *L. crustorum* MN047 showed probiotic characteristics.

## GENOME ANNOUNCEMENT

*Lactobacillus crustorum*, one of the less studied lactobacilli, was first isolated from koumiss, which is a traditional mare’s milk product fermented by its own natural microbes, including lactic acid bacteria (LAB) and yeast. For centuries, koumiss has been considered not only a wholesome beverage (1) but also a functional food for medical purposes (2, 3). LAB are recognized to play a major role in the formation of substances beneficial to human health (4). Our previous study found that the *L. crustorum* MN047 can produce bacteriocin MN047 A (BMA) (5). The BMA had broad spectrum antibacterial activity in both Gram-positive and Gram-negative pathogens, including multidrug-resistant strains. Although two genomic shotgun sequences have been assembled for *L. crustorum* (https://www.ncbi.nlm.nih.gov/genome/genomes/15930), no complete genome sequence of the species is available. To further investigate *L. crustorum* MN047 at the genetic level, this strain was subjected to complete-genome sequencing analysis.

The genome of *L. crustorum* MN047 was extracted at the log phase and constructed *de novo* by a combined strategy of both PacBio RS II and Illumina HiSeq 4000 sequencing. A 20-kb single-molecule real-time (SMRT) bell library was prepared from sheared genomic DNA using a 20-kb template library preparation workflow (6). SMRT sequencing was conducted on a PacBio RS II platform using the C4 sequencing chemistry and P6 polymerase with a 240-min movie time on one SMRT cell. Raw sequences were filtered, and 766,898,300 bp of clean data, with a total of 123,121 sequences and a mean read length of 6,228 bp, were obtained. The filtered subreads were* de novo* assembled using RS_HGAP Assembly version 3 in SMRT Analysis version 2.3.0 software (Pacific Biosciences, USA) (7), which yielded three contigs with 331-fold coverage. Correction of the PacBio assembly was performed by soapSNP and soapIndel software using data from the Illumina HiSeq 4000 sequencing. For Illumina HiSeq 4000 sequencing, a 270-bp insert library was constructed with a read length of 2 × 150 bp. After filtering for quality, 623 Mb of clean data were obtained. Finally, a chromosome and two plasmids were achieved with 0 gaps and single-base quality of 0.9951. Gene prediction was managed with Glimmer version 3.02. Annotation was performed using BLAST with the NR, Swiss-Prot, TrEMBL, COG, KEGG, and GO databases.

The genome sequence of *L. crustorum* MN047 consists of a circular chromosome (2,261,471 bp) and two circular plasmids (46,788 bp and 8,220 bp), with mean G+C contents of 35.02%, 37.91%, and 36.19%, respectively. There are 2,218 protein-coding genes, 55 tRNA genes, 12 rRNA genes, and three sRNA genes. A total of 2,176 genes were assigned a putative function, including acid tolerance, bile salt hydrolysis, cell adhesion, thiamine, riboflavine, cobalamin and folate biosynthesis, antioxidative property, heavy-metal degradation, aromatic-compounds degradation, exopolysaccharide production, and bacteriocin production. Therefore, this genome sequence provides bases for studying the mechanisms for the probiotic properties of *L. crustorum* MN047 systematically.

### Accession number(s).

The complete genome sequence of *L. crustorum* MN047 was deposited in GenBank under the accession numbers CP017996 (chromosome), CP017997 (plasmid 1), and CP017998 (plasmid 2).
